# Trajectories of opioid use among patients with low back pain: Association to work absence

**DOI:** 10.1002/ejp.4706

**Published:** 2024-08-02

**Authors:** Johan Liseth Hansen, Knut Reidar Wangen

**Affiliations:** ^1^ University of Oslo Oslo Norway; ^2^ Quantify Research Stockholm Sweden

## Abstract

**Background:**

Low back pain (LBP) is a leading reason for opioid use and a closer examination of opioid use and productivity losses among these patients is needed. We identify opioid use trajectories using a group‐based trajectory model (GBTM) and estimate productivity losses across the trajectories.

**Methods:**

Patients diagnosed with LBP in Swedish specialty care between 2011 and 2015, between the ages of 20 and 60, were included. Two GBTMs were estimated on monthly opioid use (converted to oral morphine equivalents) during the two 12‐month periods preceding and following diagnosis. Productivity losses were estimated using the human‐capital approach.

**Results:**

In total, 147,035 patients were included. The mean age at diagnosis was 43 years of age and 49% of the patients were male. A qualitative assessment of the identified groups in the GBTM models was made based on the patterns of opioid use. We chose three pre‐diagnosis groups characterized as ‘Pre‐low’ (*N* = 109,492), ‘Pre‐increase’ (*N* = 27,336) and ‘Pre‐high’ (*N* = 10,207). Similarly, four post‐diagnosis groups were chosen and characterized as ‘Post‐low’ (*N* = 73,287), ‘Post‐decrease’ (*N* = 39,446), ‘Post‐moderate’ (*N* = 20,001) and ‘Post‐high’ (*N* = 13,595). Only 50% of the patients in the ‘Pre‐high’ group were in the ‘Post‐high’ group. The total productivity losses by the pre‐diagnosis groups were more than 2.7 billion Euros over the total 6‐year study period.

**Conclusion:**

This study highlights how patients with LBP and high use of opioids are highly correlated before and after diagnosis. Patients with high use of opioids also exhibit high work absence and productivity losses.

**Significance Statement:**

This was the first study to estimate trajectories of opioids in the two time periods before and after a diagnosis of low back pain. For the first time, productivity losses were also estimated across the identified opioid use trajectories.

## 1 INTRODUCTION

Over the last few decades, the use of opioids has been increasingly scrutinized and debated. The ongoing opioid epidemic in the United States, which is estimated to have taken almost 110,000 lives in 2021—the highest number of deaths since 2000 (Spencer et al., 2022)—along with the poor outcomes stemming from long‐term use (Baldini et al., 2012; Kotlińska‐Lemieszek & Żylicz, 2022), exemplify these issues. High use of opioids is a substantial policy and clinical challenge that needs to be properly addressed.

One common underlying reason for opioid use is to seek pain relief from low back pain (LBP). According to the Global Burden of Disease Study, LBP was the leading cause of disability worldwide (Vos et al., 2017). Over 600 million people worldwide are estimated to be affected by LBP and this number is projected to rise to almost 850 million by 2050 (Ferreira et al., 2023).

People affected with LBP are often in working age and previous cost‐of‐illness studies of LBP have demonstrated relatively higher costs associated with productivity losses compared to healthcare costs (Ekman et al., 2005; Olafsson et al., 2018; Wieser et al., 2011). Another part of the literature has focused on longitudinal heterogeneity of prescription opioid use among patients with LBP and related labour market outcomes (such as sick leaves or early retirement due to disability). In a recent study, Di Donato et al. used a group‐based trajectory model (GBTM) (Nagin, 2005)—a semi‐parametric, data‐driven approach to identify longitudinal clusters or trajectories—to study opioid use after an episode of LBP among an Australian patient population (Di Donato et al., 2022). They identified three distinct trajectory groups and found that higher use of opioids after the LBP episode and longer work absences were highly correlated.

We know from previous studies of other patient groups that pre‐diagnosis opioid use is highly associated with post‐diagnosis opioid use (Goesling et al., 2016; Hansen, Heilig, et al., 2023). However, Di Donato et al. did not consider pre‐LBP episode opioid use and the cost‐of‐illness studies cited above did not examine costs across opioid use groups. In our study, we aim to address these limitations in the existing literature. We utilize comprehensive Swedish administrative data to estimate trajectories of opioid use before and after an LBP diagnosis. Our analysis includes a description of the association between pre‐ and post‐diagnosis opioid use, as well as estimates of productivity losses across different trajectory groups.

## 2 METHODS

We used Swedish nationwide and population‐wide administrative data with complete coverage for this study (see Table 1 on the registers, content and use case). Patients were identified in Swedish speciality care in the years 2008–2015 and included those with a primary diagnosis of low back pain (ICD‐10 codes: M48.0, M51, M53.2, M53.3, M53.8, M53.9, M54, M96.1) in either out‐ or inpatient hospital care. The ICD‐10 codes span a broad definition of low back pain, including dorsalgia, disc damage and spinal stenosis.

**TABLE 1:** Data sources.

Official name of register	Description and use
Swedish National Patient Register	Specialty care register covering outpatient visits and inpatient hospitalizations
	Used for identification of patients and the index date as well as comorbidities (ICD‐10 diagnosis codes)
Swedish Prescribed Drug Registry	Register with all pharmacy‐dispensed prescriptions and include data on date, ATC‐code and daily defined doses (DDD) for all prescriptions
	Data on opioid use stem from this register. Over‐the‐counter medication and medications provided directly at hospitals are not part of this register
MIDAS register	Start and end dates, and extent of absence for days on sick leave and disability pension. Sick leave are days absent from work due to sickness and disability pension are early retirement due to disability
	In the reporting of results, net absent days are reported as the number of gross days absent are multiplied by the extent of leave
	The register only register sick leaves longer than 14 days, therefore sick leaves shorter than 14 days are not captured by the register. This is an underlying limitation of the data. In Sweden, the employer covers the costs for the first 14 days of sick leave, and then the government will pay for the days beyond. Thus, the register only register the government paid sick leave periods. For the periods that are included in the register, the start date of the employer paid period is included
LISA and population register	Education and disposable income are registered on an annual calendar year basis
	Population size in the patient's home municipality on an annual basis
Swedish cause of death register	Date of death

The index date was defined as the first diagnosis date. Group‐based trajectories were defined in two distinct periods: 1 year preceding the index date (pre‐diagnosis trajectory period) and 1 year following the index date (post‐diagnosis trajectory period). Additionally, we defined a pre‐diagnosis and a post‐diagnosis period, each spanning 3 years. The study periods are illustrated in Figure S1. We wanted to study patients visiting speciality care for the first recorded time due to LBP, thus patients with a diagnosis between 2008 and 2010 were removed because we would be lacking necessary pre‐diagnosis data (i.e., including one or more years from 2005 to 2007) for study analysis. Further, we restricted the sample to patients in working age, 20–60 years at the index date.

The methods section is organized as follows: an overview of the data sources, a description of the GBTM to identify the longitudinal patterns of opioids, the regression analyses, and a description of the productivity loss estimation.

### 2.1 Data sources

Patients were linked together on an individual level between the data sources. The Swedish registers include complete coverage for the entire population without loss to follow‐up such as changing insurance providers. We were, therefore, able to identify all patients with LBP in specialty care including their medical history (prescriptions, comorbidities and healthcare visits) and socioeconomic status (disposable income and education). A more detailed description of the data sources has been published elsewhere (Hallberg et al., 2023). Ethical approval was obtained from the Stockholm Ethical Review Board for the collection and use of the data (2018/1634‐32/2).

### 2.2 Longitudinal patterns of opioid use and group‐based trajectories

The main aim of this study was to model pre‐ and post‐diagnosis opioid use trajectories separately. We applied two group‐based trajectory models (Nagin, 2005): one for the 1‐year pre‐diagnosis trajectory period and another for the 1‐year post‐diagnosis trajectory period. Subsequently, we examined the outcomes in the respective 3‐year pre‐ and post‐periods. Given that we considered the two time periods to be distinct, we chose not to estimate a combined GBTM over both pre‐ and post‐diagnosis time periods. This contrasts with the design in a previous Swedish paper applying the GBTM on sick leave, a different outcome, before and after opioid initiation among patients with chronic pain (Lalic et al., 2019). We therefore extended the findings of the Di Donato et al. study (Di Donato et al., 2022), who estimated a GBTM of opioid use after a worker compensation time loss claim due to LBP, by also estimating the pre‐diagnosis trajectories.

The input parameter and the variable of interest in the GBTM was prescription opioid use (all prescriptions with an ATC code starting with N02A) per month. Therefore, each of our two 1‐year trajectory periods included 12 time points. The total daily defined doses (DDDs) in each prescription were converted to oral morphine equivalents (OMEQs) to standardize the analgesic potency of all opioid prescriptions. The conversion ratios between different opioids were based on previous literature and treatment guidelines in the Nordics (Svendsen et al., 2012; The Association for the Publication of the Norwegian Drug Handbook, 2019; The Danish Health Data Authority, 2020), and is further described in Table S1. The data contained information on the total OMEQ in each prescription but not for how long those doses were supposed to last. To calculate a daily OMEQ dosage, we assumed that each prescription lasted for 90 days and divided the total OMEQs in each prescription by 90. This is in line with Swedish prescribing regulations stating that a patient should only be prescribed a volume covering a maximum of 90 days at the time (The Dental and Pharmaceutical Benefits Agency, 2023). We then summarized the daily OMEQ doses in each prescription by each month for the 12 months pre‐ and post‐diagnosis.

We used the Stata package ‘traj’ to run the group‐based trajectory models. For a given number of groups, we ran all possible combinations of the trajectory shapes (only intercept, linear, quadratic or cubic) each group could take over the course of the trajectory period (either pre or post). These trajectory shapes were evaluated using Bayesian information criterion (BIC) values and the one with the maximum BIC value was chosen as the main model for the given group number (Tables S3 and S4). For each number of groups between 3 and 6, we repeated this process and thus obtained four main models. The optimal number of groups appeared to be a trade‐off, as a higher number of groups tended to improve the BIC value while reducing the interpretability of the identified trajectories. While we could run models with a higher number of groups, we chose to limit ourselves to a maximum of 6 to contain the qualitative interpretation of each group. By definition, all patients were alive during the first pre‐index trajectory period. To account for death post‐diagnosis, only patients who were alive at the end of the 1‐year post‐diagnosis trajectory analysis were included in any of the post‐diagnosis trajectory groups. We therefore excluded patients who died during the post‐diagnosis trajectory period from inclusion in the post‐diagnosis GBTM.

### 2.3 Association between pre‐ and post‐diagnosis trajectory groups

We wanted to test the strength of the statistical association between pre‐diagnosis trajectory groups and post‐diagnosis trajectories in two multinomial logistic regression models, one crude and one adjusted. The outcome variable in these models was post‐diagnosis group belonging, using the ‘Post‐low’ group as the reference category. In the crude model, we included only pre‐diagnosis groups as covariates, while in the adjusted model we also added patient characteristics as covariates. Opioid use itself was not included in the models, as the pre‐diagnosis groups belonging captured the effect of opioids. For this analysis, we did not aim to produce the highest predictive model or right‐hand side variables based on fit criteria, rather to test simple statistical associations.

### 2.4 Productivity losses

We estimated productivity losses using the human‐capital approach (Krol & Brouwer, 2014; Weisbrod, 1961). In the human‐capital approach, the value of each day absent from work is assumed to be equal to each individual's marginal productivity. This is traditionally measured as the daily wage rate. In our dataset, the individual daily wage rate was available (annual disposable income divided by 365). Therefore, our productivity loss estimation per day was as follows:

$${\text{Productivity loss}}_{\mathrm{it}}={\text{wage}}_{i}\cdot {\text{absence}}_{\mathrm{it}}$$

${\text{wage}}_{i}$ is the individual daily wage rate in the calendar year preceding the index date and ${\text{absence}}_{\mathrm{it}}$ was equal to 1 if the individual *i* was absent from work at day *t* due to sick leave or disability pension as recorded by the registers. The daily productivity loss was then summarized over the two 3‐year periods before and after diagnosis.

## 3 RESULTS

The results section is organized as follows: a description of the overall study population, the construction of the opioid use groups by the GBTM, a description of the GBTM‐identified opioid use groups, the regression analyses, followed by presentation of the productivity loss estimates.

### 3.1 Study population

Almost 150,000 patients were identified with their first LBP diagnosis between 2011 and 2015 in Swedish speciality care. Among all patients in the sample, 49% were males and the mean age at diagnosis was 43 years (Table 2). Over 30% had additional education beyond upper secondary school and almost 7% of patients received disability pension and were permanently outside the labour market. The average disposable income was 27,408 Euro. Half of the patients had a recorded diagnosis of another chronic pain‐related condition than low back pain.

**TABLE 2:** Patient characteristics at index among all patients in column 2, and stratified by pre‐diagnosis trajectory groups (columns 3–5) and post‐diagnosis groups (columns 6–9).

Characteristic	All patients		Pre‐diagnosis groups			Post‐diagnosis groups		
	Overall, *N* = 147,035	Pre‐low, *N* = 109,492	Pre‐increase, *N* = 27,336	Pre‐high, *N* = 10,207	Post‐low, *N* = 73,287	Post‐decrease, *N* = 39,446	Post‐moderate, *N* = 20,001	Post‐high, *N* = 13,595
Age	43 (11)	42 (11)	43 (10)	45 (10)	42 (11)	42 (10)	43 (11)	45 (10)
Males	71,434 (49%)	54,569 (50%)	12,578 (46%)	4287 (42%)	35,282 (48%)	20,782 (53%)	8999 (45%)	5993 (44%)
Education								
<Upper secondary school	34,983 (24%)	24,190 (23%)	7293 (27%)	3500 (35%)	15,875 (22%)	8748 (23%)	5550 (28%)	4579 (34%)
Upper secondary school	64,905 (45%)	47,909 (45%)	12,277 (46%)	4719 (47%)	31,768 (44%)	17,707 (46%)	8875 (45%)	6243 (46%)
>Upper secondary school	44,644 (31%)	35,348 (33%)	7408 (27%)	1888 (19%)	24,259 (34%)	12,290 (32%)	5295 (27%)	2651 (20%)
Employment status								
At disability pension	9859 (6.7%)	5820 (5.3%)	1833 (6.7%)	2206 (22%)	4108 (5.6%)	1745 (4.4%)	1519 (7.6%)	2314 (17%)
Employed	113,645 (77%)	86,593 (79%)	21,173 (77%)	5879 (58%)	57,572 (79%)	32,089 (81%)	15,028 (75%)	8565 (63%)
Not employed	23,429 (16%)	16,986 (16%)	4322 (16%)	2121 (21%)	11,548 (16%)	5578 (14%)	3450 (17%)	2711 (20%)
Disposable income (EUR)	27,408 (30,929)	28,103 (29,474)	26,499 (38,356)	22,401 (22,109)	28,103 (29,685)	28,392 (37,296)	26,121 (26,861)	22,966 (20,894)
Population density of home municipality								
High	19,283 (13%)	15,063 (14%)	3299 (12%)	921 (9.0%)	10,314 (14%)	5100 (13%)	2521 (13%)	1306 (9.6%)
Low	107,704 (73%)	79,349 (72%)	20,343 (74%)	8012 (78%)	52,808 (72%)	28,966 (73%)	14,661 (73%)	10,642 (78%)
Medium	20,048 (14%)	15,080 (14%)	3694 (14%)	1274 (12%)	10,165 (14%)	5380 (14%)	2819 (14%)	1647 (12%)
Number of sick days	54 (143)	39 (120)	80 (158)	153 (242)	46 (129)	38 (114)	69 (156)	120 (218)
Number of disability days	83 (268)	66 (241)	86 (269)	264 (425)	70 (247)	56 (222)	95 (282)	210 (394)
Number of outpatient visits	6 (10)	5 (9)	7 (12)	12 (16)	6 (10)	5 (8)	7 (11)	10 (14)
Number of hospitalization days	3.6 (22.7)	2.7 (21.3)	4.5 (23.1)	10.1 (32.4)	3.0 (23.7)	2.4 (13.4)	4.2 (23.6)	8.0 (30.1)
Total OMEQ–opioids	2644 (18,445)	163 (1398)	1835 (4837)	31,424 (62,621)	281 (3179)	834 (7545)	1544 (5620)	21,769 (54,200)
Total DDD–NSAIDs	79 (194)	51 (144)	125 (213)	248 (395)	53 (145)	64 (154)	112 (224)	206 (353)
Total DDD–paracetamol	50 (155)	23 (88)	73 (148)	282 (376)	25 (90)	33 (103)	66 (153)	213 (341)
Total DDD–other analgesics	67 (323)	38 (231)	86 (354)	324 (719)	35 (217)	48 (259)	87 (358)	260 (655)
Type of LBP diagnosis								
Dorsalgia	120,179 (82%)	93,203 (85%)	18,815 (69%)	8161 (80%)	61,365 (84%)	32,357 (82%)	15,236 (76%)	10,571 (78%)
Intervertebral disc damage	21,220 (14%)	12,783 (12%)	7078 (26%)	1359 (13%)	9311 (13%)	6234 (16%)	3474 (17%)	2170 (16%)
Spinal stenosis	5636 (3.8%)	3506 (3.2%)	1443 (5.3%)	687 (6.7%)	2611 (3.6%)	855 (2.2%)	1291 (6.5%)	854 (6.3%)
Any chronic pain related	74,408 (51%)	51,468 (47%)	15,595 (57%)	7345 (72%)	35,323 (48%)	16,177 (41%)	12,508 (63%)	9896 (73%)
Multimorbidities	1431 (1.0%)	877 (0.8%)	288 (1.1%)	266 (2.6%)	586 (0.8%)	251 (0.6%)	236 (1.2%)	330 (2.4%)
Intervertebral disc disorder	8186 (5.6%)	5088 (4.6%)	2008 (7.3%)	1090 (11%)	3439 (4.7%)	1738 (4.4%)	1522 (7.6%)	1438 (11%)
Other musculoskeletal conditions	29,515 (20%)	19,262 (18%)	6604 (24%)	3649 (36%)	13,500 (18%)	5362 (14%)	5553 (28%)	5034 (37%)
Migraine and headaches	9034 (6.1%)	5912 (5.4%)	2062 (7.5%)	1060 (10%)	3984 (5.4%)	1989 (5.0%)	1730 (8.6%)	1313 (9.7%)
Arthritis	17,491 (12%)	12,702 (12%)	3116 (11%)	1673 (16%)	10,143 (14%)	2638 (6.7%)	2574 (13%)	2108 (16%)
Cancer diagnosis	7836 (5.3%)	5198 (4.7%)	1736 (6.4%)	902 (8.8%)	3383 (4.6%)	1621 (4.1%)	1251 (6.3%)	1171 (8.6%)
Other chronic pain conditions	37,635 (26%)	24,925 (23%)	8276 (30%)	4434 (43%)	16,805 (23%)	8184 (21%)	6643 (33%)	5802 (43%)
Death within 1 year	706 (0.5%)	367 (0.3%)	185 (0.7%)	154 (1.5%)	0 (0%)	0 (0%)	0 (0%)	0 (0%)
Death within 3 years	1609 (1.1%)	866 (0.8%)	399 (1.5%)	344 (3.4%)	253 (0.3%)	157 (0.4%)	183 (0.9%)	310 (2.3%)

*Note*: The detailed definitions of all variables are found in Table S2. All numeric table entries are either Mean (Standard deviation) or Number (Percent).

**FIGURE 1:**
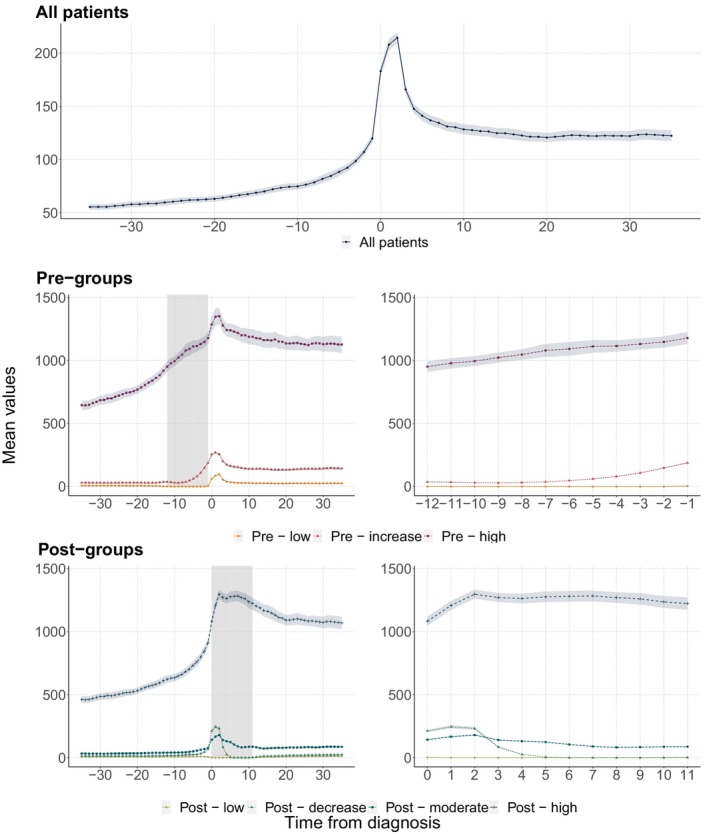
Longitudinal patterns of opioid use among all patients and the trajectory groups. The trajectory groups were estimated based on the sum of OMEQs. The shaded areas in the left panels on rows 2 (pre‐diagnosis) and 3 (post‐diagnosis) are the time periods where the GBTM was applied. The right panels in rows 2 and 3 show opioid use during the shaded trajectory periods with higher resolution.

**FIGURE 2:**
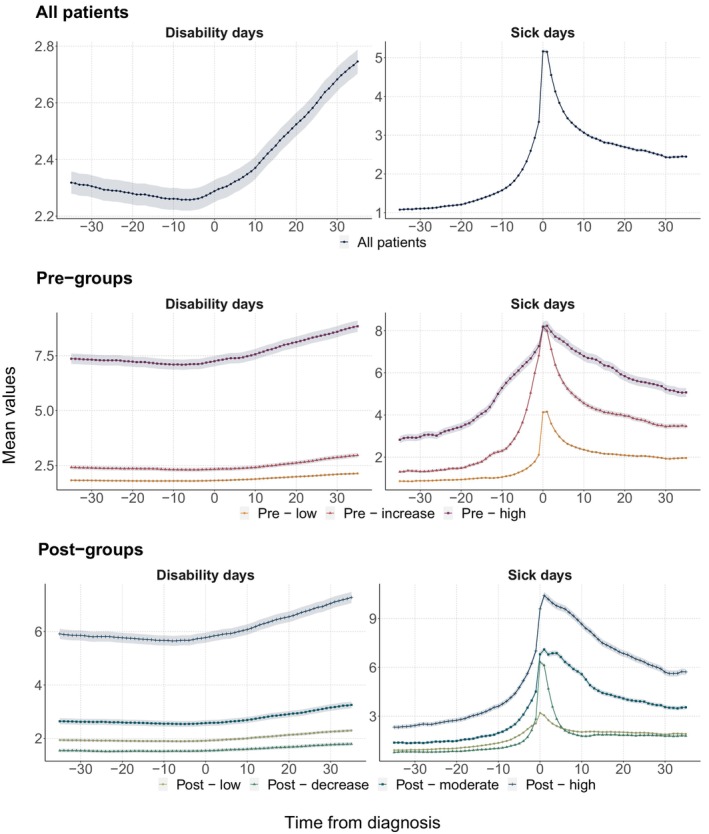
Developments in disability and sick days among all patients (row 1) and the trajectory groups identified pre‐diagnosis (row 2) and post‐diagnosis (row 3).

Figure 1 this figure presents the opioid use among all patients. The average opioid use increased steadily up to the time of diagnosis among all patients where it peaked at more than 200 OMEQs a month. About 5 months after diagnosis, opioid use stabilized at around 125 OMEQs per month, which was substantially higher than pre‐diagnosis use. Figure 2 presents the average number of disability days and sick days among all patients. For sick days, the pattern has similarities with the pattern for opioid use, with a peak at 5 sick days in the month of diagnosis and higher levels after the diagnosis than before. For disability days (Figure 2), the number of days decreased moderately in the months leading up to diagnosis, followed by a steeper increase that continued to the end of the study period.

### 3.2 Longitudinal patterns of opioid use and group‐based trajectories

Longitudinal patterns of opioid use were defined and identified with a group‐based trajectory model (GBTM).

#### 3.2.1 Pre‐diagnosis trajectory groups–Construction

In the pre‐diagnosis models, greater model complexity (modelled with many cubic terms) yielded higher BIC values (Table S3). The model with the highest BIC values was the model with six pre‐specified groups. We did not estimate models with more than 6 groups. A few common patterns were observed across all models with different pre‐specified number of groups. All models had one distinct group with low levels of opioid use and one distinct group with high levels (Figure S2). When a greater number of groups was added to the model, the new groups that were identified mostly fell in between the low and high usage groups. Heterogeneity of the mid‐use groups therefore increased as more groups were added.

#### 3.2.2 Post‐diagnosis trajectory groups–Construction

In the post‐diagnosis models, the patterns were similar as for the pre‐diagnosis models. The models with six groups yielded higher BIC values (Table S4), and more groups increased heterogeneity among the mid‐use groups (Figure S2). A difference to the pre‐diagnosis trajectory was that by going from three to four groups, a more distinct high‐use group (around 1200–1300 OMEQs a month) was identified that was also identified in the models with five or six groups.

#### 3.2.3 Pre‐diagnosis trajectory groups–Description

While increasing the number of pre‐specified groups in the estimation increased model fit and identified additional groups in the data, a higher number of groups comes at a cost of reduced qualitative interpretability. To ensure qualitative interpretability, we chose to continue our analyses with three pre‐diagnosis trajectories. The most distinct group of high‐use patients, being prescribed over 1000 OMEQs a month was identified in the model with three groups (Figure S2). Adding groups did not substantially alter the trajectory with the highest use. These models added groups among the lower‐use patients. The pre‐diagnosis groups were named ‘Pre‐low’ (a group with almost no opioid use), ‘Pre‐increase’ (with increasing opioid use from low levels leading up to diagnosis) and ‘Pre‐high’ (consistently high levels of opioid use). While we did identify patients in the 1‐year pre‐diagnosis period, the patients in these groups also had very different opioid use patterns outside this identification period (Figure 1). The ‘high‐use’ group had consistently higher opioid use than the other two groups and the ‘Pre‐increase’ group also had consistently higher use than the ‘Pre‐low’ group.

Among the pre‐diagnosis trajectory groups, the ‘Pre‐high’ group had lower education, lived in more rural areas and had more chronic pain‐related comorbidities than the ‘Pre‐low’ and ‘Pre‐increase’ groups (Table 2). The ‘Pre‐high’ group had around 80% of the income level of the patients in the ‘Pre‐low’ group. They were also prescribed more non‐opioid pain analgesics compared to the other groups. The ‘Pre‐increase’ group was generally in between the ‘Pre‐low’ and ‘Pre‐high’ groups across all outcomes.

The longitudinal patterns of sick and disability days differed between the pre‐diagnosis trajectory groups (Figure 2). The high‐use group consistently had the highest number of sick and disability days also outside the 1‐year pre‐diagnosis trajectory period. Common for all pre‐diagnosis groups was an increase in the number of sick days leading up to diagnosis, before the number of sick days decreased towards a higher level than before diagnosis. The ‘Pre‐increase’ group had a greater spike in sick days than all the other groups, and in the months around diagnosis had as many sick days as the ‘Pre‐high’ group, but not in the months further away from the diagnosis month. The trend in disability days was different than for sick days and we did not observe the same spike around the time of diagnosis. Across all pre‐groups, a flat or slightly decreasing trend was observed before diagnosis. After diagnosis, the number of disability days increased until the end of the study period. This trend was the strongest for the ‘Pre‐high’ group.

#### 3.2.4 Post‐diagnosis trajectory groups–Description

By the same qualitative interpretation rationale as for the pre‐diagnosis group, we chose to continue with four post‐diagnosis trajectories. The model with 4 groups identified a more distinct high‐use group than that with three groups. The other trajectories were characterized by almost no opioid use (the ‘Low’ group), a ‘Moderate’ (with a relatively constant, but moderate level of opioid use, around 100 monthly OMEQ's) and a ‘Decrease’ group (started at the same level as the ‘Moderate’ group, but decreased over time towards the ‘Low’ group). As was the case for the ‘Pre‐high’ group, the ‘Post‐high’ group had consistently higher use of opioid use than the other groups, also outside the identification period (Figure 1). Outside the trajectory period, the ‘Post‐moderate’ group had the second highest level of opioid use.

The ‘Post‐low’ group had the highest levels of education, employment and income, as well as the least comorbidities of all groups (Table 2—columns 6–9). As with the ‘Pre‐high’ group, the high‐use group had the lowest socioeconomic status and the most comorbidities compared to the other groups.

Sick leave and disability patterns, stratified by the post‐diagnosis groups, reveal the same patterns as for the pre‐diagnosis groups (Figure 2). The ‘Post‐decrease’ and ‘Post‐moderate’ had higher peaks of sick leave around the time of diagnosis than the ‘Post‐low’ group, but not as high as the ‘Post‐high’ group. For disability days, the level of leave was different across the groups, but all groups saw an increase after diagnosis.

### 3.3 Association between pre‐ and post‐diagnosis trajectory groups

#### 3.3.1 Transitions between pre‐ and post‐diagnosis groups

Table 3 presents the transitions or flows from the pre‐diagnosis groups to the post‐diagnosis groups. Around 74% (109,492/147,035 = 0.744) of patients were in the ‘Pre‐low’ group and 50% (73,287/147,035 = 0.498) of patients were in the ‘Post‐low’ group (of all patients, including the deceased post‐diagnosis). This indicates a greater spread of patients across opioid use groups in the post‐diagnosis period. Of the patients in the ‘Post‐high’ group, almost 20% (2648/13,595 = 0.194) were in the ‘Pre‐low’ group and 52% came from the ‘Pre‐high’ group. Almost 90% of patients in the ‘Post‐low’ group were in the ‘Pre‐low’ group and only 0.8% were in the ‘Pre‐high’ group.

**TABLE 3:** Transition figures from pre‐diagnosis groups to post‐diagnosis groups. The column sums refer to the composition of the post‐diagnosis status.

	Death	Post‐low	Post‐decrease	Post‐moderate	Post‐high	Sum
Pre‐low	367	64,342	30,049	12,086	2648	109,492
Pre‐increase	185	8400	8499	6380	3872	27,336
Pre‐high	154	545	898	1535	7075	10,207
Sum	706	73,287	39,446	20,001	13,595	147,035

Patients who died during the 1‐year post‐diagnosis period, 0.5% of patients, were not part of the GBTM estimation of post‐trajectory groups. The largest pre‐group among the deceased patients in absolute numbers was the ‘Pre‐low’ group (52%) and 22% were in the ‘Pre‐high’ group. There was a low number of deaths; however, there was a difference between the ‘Pre‐High’ group and the other groups in terms of post‐index survival curves (Kaplan–Meier estimates in Figure S3).

#### 3.3.2 Regression results

As observed in the descriptive results above, the groups with higher opioid use had lower socioeconomic status and were sicker (in terms of number of comorbidities and non‐opioid analgesic medication use) than the other groups were. To test the statistical strength of these associations, we estimated two multinomial logistic regression models with the post‐diagnosis trajectory group as the outcome.

The odds ratios for pre‐diagnosis groups were higher in the crude model compared to the adjusted model. For example, the odds ratio for being in the ‘Post‐high’ group when being in the ‘Pre‐high’ group compared to the ‘Pre‐low’ group decreased from 315 in the crude model to 138 in the adjusted model.

In the adjusted model, the largest odds ratios were still for the pre‐diagnosis trajectory groups, indicating the strong association of the pre‐diagnosis groups to post‐diagnosis groups compared to the ‘Pre‐low’ reference group (Table 4). A higher socioeconomic status (higher education, employment, higher disposable income) was also associated with a lower odds of belonging to the ‘Post‐high’ group compared to the ‘Post‐low’ group.

**TABLE 4:** Predictors of post‐diagnosis trajectory groups (crude and adjusted model). Numerical variables (except age) were log‐transformed.

	Post‐decrease			Post‐moderate			Post‐high		
Characteristic	OR	95% CI	*p*‐value	OR	95% CI	*p*‐value	OR	95% CI	*p*‐value
Crude model									
Pre‐diagnosis groups									
Pre‐low	–	–	–	–	–	–	–	–	–
Pre‐increase	2.17	2.10, 2.24	<0.001	4.04	3.89, 4.20	<0.001	11.2	10.6, 11.8	<0.001
Pre‐high	3.53	3.17, 3.93	<0.001	15.0	13.6, 16.6	<0.001	315	287, 347	<0.001
Adjusted model									
Pre‐diagnosis groups									
Pre‐low	–	–	–	–	–	–	–	–	–
Pre‐increase	1.99	1.92, 2.06	<0.001	2.72	2.61, 2.83	<0.001	6.86	6.46, 7.28	<0.001
Pre‐high	3.31	2.96, 3.69	<0.001	8.68	7.82, 9.63	<0.001	138	125, 152	<0.001
Age	1.00	1.00, 1.00	0.100	1.00	0.99, 1.00	<0.001	1.00	1.00, 1.00	0.046
Male	1.14	1.11, 1.17	<0.001	1.02	0.99, 1.06	0.200	1.18	1.12, 1.24	<0.001
Education									
<Upper secondary school	–	–	–	–	–	–	–	–	–
Upper secondary school	0.98	0.95, 1.02	0.300	0.87	0.83, 0.91	<0.001	0.83	0.79, 0.88	<0.001
>Upper secondary school	0.91	0.88, 0.94	<0.001	0.73	0.70, 0.76	<0.001	0.61	0.57, 0.66	<0.001
Employment status									
At disability pension	–	–	–	–	–	–	–	–	–
Employed	1.35	1.27, 1.44	<0.001	1.09	1.01, 1.17	0.024	0.84	0.77, 0.92	<0.001
Not employed	1.10	1.03, 1.18	0.007	1.06	0.98, 1.15	0.200	0.88	0.80, 0.97	0.011
Disposable income (EUR)	1.00	0.98, 1.01	0.400	0.98	0.97, 1.00	0.015	0.96	0.94, 0.98	<0.001
Population density	1.00	1.00, 1.01	0.400	1.01	1.00, 1.01	0.008	1.00	0.99, 1.01	0.800
Sick days	0.95	0.94, 0.96	<0.001	0.99	0.98, 1.00	0.027	0.99	0.98, 1.01	0.300
Outpatient visits	0.86	0.84, 0.87	<0.001	0.97	0.95, 0.99	<0.001	0.85	0.83, 0.88	<0.001
Hospitalization days	1.04	1.02, 1.06	<0.001	1.03	1.01, 1.05	<0.001	1.07	1.05, 1.10	<0.001
DDD‐NSAIDs	1.06	1.05, 1.07	<0.001	1.13	1.12, 1.14	<0.001	1.11	1.10, 1.13	<0.001
DDD‐paracetamol	1.06	1.06, 1.07	<0.001	1.09	1.08, 1.10	<0.001	1.17	1.16, 1.19	<0.001
DDD‐other pain medications	1.05	1.04, 1.06	<0.001	1.07	1.06, 1.08	<0.001	1.14	1.12, 1.15	<0.001
Type of LBP diagnosis									
Dorsalgia	‐	‐	‐	‐	‐	‐	‐	‐	‐
Intervertebral disc damage	1.00	0.96, 1.04	>0.900	1.14	1.08, 1.19	<0.001	1.02	0.95, 1.09	0.600
Spinal stenosis	0.58	0.53, 0.63	<0.001	1.56	1.45, 1.69	<0.001	1.15	1.03, 1.28	0.014
Multimorbidities	0.95	0.81, 1.10	0.500	1.23	1.04, 1.45	0.013	1.73	1.42, 2.10	<0.001
Intervertebral disc disorder	1.03	0.97, 1.09	0.400	1.29	1.20, 1.38	<0.001	1.40	1.28, 1.52	<0.001
Other musculoskeletal conditions	0.79	0.76, 0.82	<0.001	1.47	1.41, 1.53	<0.001	1.86	1.76, 1.96	<0.001
Migraine and headaches	1.01	0.95, 1.07	0.800	1.32	1.24, 1.41	<0.001	1.17	1.07, 1.28	<0.001
Arthritis	0.52	0.50, 0.55	<0.001	0.79	0.75, 0.83	<0.001	0.79	0.73, 0.85	<0.001
Cancer	1.02	0.95, 1.08	0.600	1.24	1.15, 1.33	<0.001	1.53	1.39, 1.68	<0.001

Abbreviations: CI, Confidence Interval; OR, Odds Ratio.

### 3.4 Productivity losses

The average productivity losses due to sick leave per patient were the lowest in the ‘Pre‐low’ group (2431 Euro vs. 8.930 Euro in the ‘Pre‐high’ group) in the 3 years before diagnosis (Table 5). For disability pension costs, the losses were 2947 Euro in the ‘Pre‐low’ group and 12,156 Euro in the ‘Pre‐high’ group in the same time period. Individual‐level productivity losses therefore reflected the patterns of sick leave and disability across all groups and time periods.

**TABLE 5:** Productivity losses in Euros.

Group	Sick leave costs		Disability leave costs	
	Before diagnosis	After diagnosis	Before diagnosis	After diagnosis
Pre‐diagnosis groups				
Pre‐low	2431 (9187) [265,937,730]	5820 (15,055) [636,677,633]	2947 (12,491) [322,388,508]	3316 (13,872) [362,784,217]
Pre‐increase	5102 (13,258) [139,405,055]	10,627 (19,147) [290,380,891]	3811 (13,088) [104,126,916]	4366 (14,665) [119,313,672]
Pre‐high	8930 (15,134) [91,141,627]	13,952 (22,382) [142,391,569]	12,156 (22,680) [124,064,110]	13,650 (24,206) [139,309,864]
Post‐diagnosis groups				
Post‐low	2874 (8600) [210,412,155]	5113 (13,623) [374,387,100]	3121 (13,001) [228,544,883]	3538 (14,008) [259,062,649]
Post‐decrease	2424 (9932) [95,539,531]	5636 (13,654) [222,122,415]	2473 (10,764) [97,480,063]	2832 (13,151) [111,598,947]
Post‐moderate	4300 (11,466) [85,977,126]	11,526 (19,802) [230,470,971]	4321 (14,551) [86,404,102]	4954 (16,220) [99,054,341]
Post‐high	7065 (15,181) [96,012,619]	17,542 (26,767) [238,397,638]	9624 (20,622) [130,784,699]	11,064 (22,188) [150,365,910]

*Note*: Individual mean (standard deviation) [Sum of all individuals]. Only patients with income data were included in the productivity loss estimations.

The total losses (the sum of all individuals of both sick leave and disability leave costs) among the pre‐diagnosis groups were the largest among the ‘Pre‐low’ group with combined costs of 1.58 billion Euros (the sum across all four columns in Table 5). This is due to the large number of patients in the ‘Pre‐low’ group compared to the other groups. The ‘Pre‐high’ group represent 7% of all patients, but accounts for 18% of all productivity losses in the pre‐diagnosis groups. Across sick leave and disability leave, the total productivity losses by the pre‐diagnosis groups were more than 2.7 billion Euros over the total 6‐year study period.

Similar cost patterns are observed for the post‐diagnosis groups, with the ‘Post‐low’ group accounting for 40% of total losses, while the ‘Post‐high’ group has the highest average per patient (7065 Euro in sick leave costs before diagnosis and 17,542 Euros after diagnosis). Over 20% of total productivity losses are attributed to the ‘Post‐high’ group, while 9.2% of patients are in this group.

## 4 DISCUSSION

We estimated two sets of GBTM in two different time periods around the first diagnosis of LBP. In each of these time periods, we identified a patient group whose opioid use was substantially greater than that of other patients with LBP. In both periods, the high opioid use groups had lower incomes, lower education and were less attached to the labour market compared to other LBP patients. If they were part of the labour market they had more sick leaves. These associations were stable over our 6‐year study period indicating problematic aspects associated with long‐term opioid use. While the high‐use patients always exhibited lower socioeconomic status, the composition of the ‘Post‐high’ group was different from the ‘Pre‐high’ group as only 50% of the ‘Post‐high’ patients came from the ‘Pre‐high’ group. This may be a problematic signal to clinicians, policymakers and researchers, as it seems like no matter when high opioid groups are identified they have lower socioeconomic status.

Lower socioeconomic status among high‐use groups may be linked to comorbidities, living and work conditions, or greater acceptability of being prescribed opioid treatment by physicians. Although no definitive causal evidence on the relationship between socioeconomic status and opioid use exists, the association between high opioid use (including opioid overdoses) and lower socioeconomic status has been previously examined in multiple countries (Altekruse et al., 2020; Böckerman et al., 2021; Nestvold et al., 2024; Serra‐Pujadas et al., 2021). Thus, while this association is not novel, our ability to reaffirm it with a data‐driven approach to identify opioid use groups adds further validation.

The explicit modelling of pre‐diagnosis opioid use and its implications is an extension of previous research, particularly the Australian Di Donato et al. study (Di Donato et al., 2022). One difference between our two study designs was the index point. Di Donato et al. followed patients from a sick leave claim due to LBP, while we followed patients from the LBP diagnosis. However, the identification of a high‐dose group, low‐use group and a moderate‐use group after the event of interest is common for both studies.

The statistical association of the pre‐diagnosis groups to the post‐diagnosis group belonging was very strong, specifically for the ‘Pre‐high’ to ‘Post‐high’ combination (odds ratio of 138 compared to ‘Post‐low’). The strong association of previous opioid use for future opioid use is in line with previous research (Goesling et al., 2016; Hansen, Heilig, et al., 2023). Opioids may have been prescribed prior to the diagnosis date in our study for multiple reasons. Patients with high pre‐diagnosis use had an additional number of pain‐related diagnoses than LBP prior to index and may have received opioids for those conditions. For example, 16% of the ‘Pre‐high’ patients had a type of arthritis compared to 12% among the ‘Pre‐low’ group. The index date was also the first speciality hospital care visit due to LBP and many patients may have been referred to a specialist from their general practitioner (GP) and prescribed opioids or other analgesics from the GP. An understanding of the patient background is crucial when treatment choices are made at the time of diagnosis by a medical specialist. Therefore, the pre‐diagnosis patterns of opioid use should not be ignored and be studied in detail when examining post‐diagnosis patterns of opioid use.

Patterns of sick leave across the trajectory groups were reflected in productivity losses per patient. Sick leave losses after diagnosis were 17,542 Euros and 5113 Euros in the ‘Post‐high’ and ‘Post‐low’ groups, respectively. The high‐use groups accounted for disproportionately large shares of total productivity losses relative to the number of patients in those groups. About 9% of patients that belonged to the ‘Post‐high’ group, accounted for 20% of the total productivity losses. The groups with the highest productivity losses before diagnosis were also the groups with the highest productivity losses after diagnosis, highlighting the strong association with the patterns of opioid use. Previous cost‐of‐illness studies of LBP have not explored these heterogeneities directly. We employed individual‐level wages which allowed us to accommodate wage heterogeneity and our estimates are not directly comparable to the Swedish LBP cost study (Olafsson et al., 2018), which used national average wages.

### 4.1 Limitations

The GBTM method has been critically evaluated for the possibility of identifying spurious relationships in the data (Mésidor et al., 2022). While this critique is valid, the trajectories we identified did not identify highly surprising patterns. A priori, we would have expected that there would be distinct groups of patients exhibiting a very high use of opioids and that a large proportion of patients would have low or no use.

The register only records state‐covered sick days, excluding sick leaves shorter than 14 days covered by the employer. This lack of data for shorter sick leave episodes is leading to an underestimation of total productivity losses. The costing method of productivity losses due to sick leave and disability pension followed the human‐capital approach; an alternative method could have been the friction cost approach. This approach may provide a greater range of estimates than the human‐capital approach (Hansen, Fast, & Wangen, 2023).

### 4.2 Future research

The two ‘High’ use groups stand out the most due to the distinct nature of their opioid use patterns. The high productivity losses in these groups warrant more focused research as they are costly to society. Additionally, some individuals exhibited noteworthy patterns, such as those in the ‘Pre‐low’ and ‘Post‐high’ groups (*N* = 2648, 20% of the ‘Post‐high’ group), who experienced a substantial increase in their exposure to opioids. Identifying patients who benefit from opioid use is important for planning and providing care and follow‐up, as well as for designing return‐to‐work policies and medical interventions. A causal understanding of the relationships between opioid use, socioeconomic status, comorbidities, pain disease characteristics and economic outcomes is outside the scope of this study. Future research could compare patients treated under different treatment regimens, such as those in countries with different approaches to opioid prescribing or before and after changes to treatment guidelines.

### 4.3 Conclusion

In both the pre‐ and post‐diagnosis periods, patients who received an LBP diagnosis can be separated into distinct groups with low‐, high or moderate levels of opioid use. A majority of patients in the post‐high use group were also in the pre‐high group and the majority of patients in the post‐low group were also in the pre‐low group. However, a notable number of patients transitioned from the pre‐low group to the post‐high group and from the pre‐high group to the post‐moderate and post‐decreasing groups. Work absence was positively associated with opioid use and the high‐use groups accounted for disproportionately large shares of the total productivity losses. Patient groups with high opioid use have lower socioeconomic status, more comorbidities and higher productivity losses than patients with lower opioid use and represent a substantial clinical and economic challenge. These findings highlight the importance of considering the heterogeneity of opioid use among patients with LBP when developing effective prevention and treatment strategies.

## AUTHOR CONTRIBUTIONS

JLH contributed to the study design, data acquisition, analysis, interpretation of data, drafting of the manuscript and critical review. KRW contributed to the study design, interpretation of data, drafting of the manuscript and critical review.

## FUNDING INFORMATION

Data holders of Swedish administrative data charge researchers for their work extracting and compiling the datasets before data are delivered to researchers. These fees were paid by Quantify Research for the data used in this study. Quantify Research received consultancy fees to extract data. All study data used is the property of the respective data holders in this study. No ownership of patient‐level data extracted from the registries can or has been transferred to anyone and the authors cannot therefore make the data available. Ultimately, the terms of use of all data are governed by the ethics and data holder approvals for this study. No other funding support was provided for the conceptualization, design, analysis or preparation of the manuscript.

## CONFLICT OF INTEREST STATEMENT

JLH is an employee of Quantify Research, a consultancy firm providing services to public and private entities, including pharmaceutical companies. KRW declare no competing interests.

## Supporting information


Data S1.
