# Supplementary material for: Trajectories of opioid use among patients with low back pain: Association to work absence
Source: Eur J Pain. 2024 Aug 2;29(1):e4706. doi: 10.1002/ejp.4706 (PMC11610708; doi:10.1002/ejp.4706)
Supplement: Supplementary file 1 — Data S1. [file EJP-29-0-s001.docx]

# Supplementary material for “Trajectories of opioid use among patients with low back pain: Association to work absence”

## Methods

Figure S1 shows an overview of all the time periods used in the study.


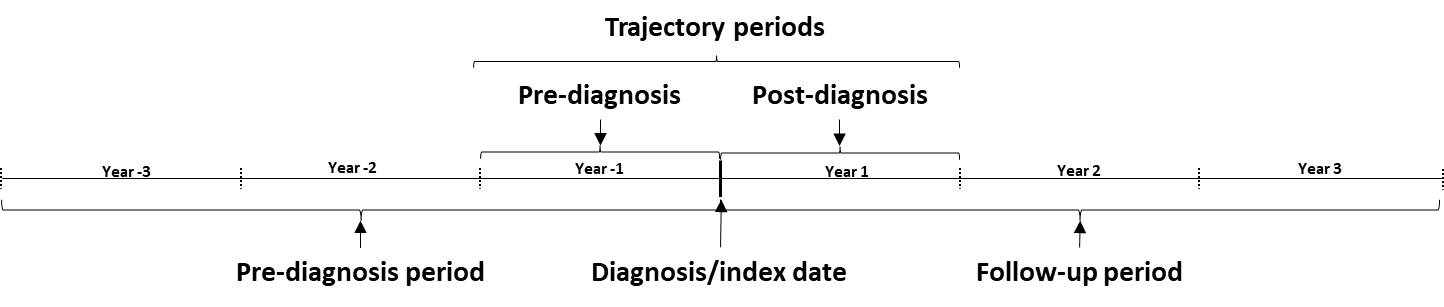


Figure S1: Study schematic

## OMEQ calculation

OMEQ’s are used in this study to model opioid use in the GBTM. This is done my multiplying the prescribed daily defined doses, recorded for each prescription, by an equianalgesic ratio to ensure the approximate same analgesic effect of the prescription. The equianalgesic ratios are different for each opioid, and depend on a range of factors such that the opioid type, release formulation, route of administration etc. The exact formula is ‘equianalgesic ratio’*’DDD in the prescription’/’DDD of morphine’. The ratios are based on previous literature and listed in Table S1 (Svendsen et al., 2012; The Association for the publication of the Norwegian Drug Handbook 2019; The Danish Health Data Authority 2020).

*Table S1: Equianalgesic ratios for different opioids*

| Opioid | ATC codes | DDD (mg) | Admin form | Equianalgesic ratios* |
| --- | --- | --- | --- | --- |
| Codeine | N02AJ06-09, | 120/90 | Oral/rectal | 0.15 |
| Tramadol | N02AX02, N02AJ13-14 | 300 | Oral | 0.2 |
| Dextropropoxyphen | N02AC04 (+ combinations N02AC54 and N02AC74) | 140 | Oral | 0.15 |
| Buprenorphin | N02AE01 | 1.2 | Sublingual | 50 |
|  |  | 1.2 | Transdermal | 100 |
| Fentanyl | N02AB03/N01AH01 | 1.2 | Transdermal | 100 |
|  |  | 0.6 | Transmucosal | 50 |
|  |  | 0.6 | Nasal | 150 |
| Hydromorphone | N02AA03 (+combinations N02AAG04) | 20 | Oral | 8 |
|  |  | 4 | Parenteral | 30 |
| Ketobemidon | N02AB01 (+combinations N02AAG02) | 50 | Oral | 1 |
|  |  | 50 | Parenteral | 3 |
| Morphine | N02AA01 (+ combinations N02AAG01) | 100 | Oral | 1 |
|  |  | 30 | Parenteral | 3 |
| Oxycodone | N02AA05 add N02AA55 (oxycodone combinations) | 75 | Oral | 1.5 |
|  |  | 30 | Parenteral | 3 |
| Tapentadol | N02AX06 | 100 | Oral | 0.3 |

## Variables

Variables presented in [Table](#tbl-predictors)S2 were used to describe patient characteristics, stratified by groups identified in the GBMT, and as predictor variables of the trajectory groups.

Table S2: Definitions of patient characteristics variables

| **Variable** | **Type** | **Definition** |
| --- | --- | --- |
| **Age** | Numeric | Age at index. |
| **Sex** | Binary | Sex (male, female) at index. |
| **Education** | Categorical | Educational level at index (<upper secondary school; upper secondary school; >upper secondary school. |
| **Employment status** | Categorical | Employment status at index (At disability pension; Employed; Not employed). |
| **Disposable income** | Numeric | Disposable income (000’s Euro) in calendar year prior to index. The variable was logged in regression analyses. The variable was inflation-adjusted from the index year to 2022, then converted to Euro from Swedish kroner. |
| **Population density** | Categorical | Population density of home municipality in year of index (low [<300 inhabitants per square kilometre], mid [300-1500], high [>1500]). |
| **Sick days** | Numeric | Number of recorded net sick leave days during the three-year pre-index period. |
| **Disability days** | Numeric | Number of recorded net disability days during the three-year pre-index period |
| **Outpatient visits** | Numeric | Number of outpatient visits during the three-year pre-index period. |
| **Hospitalization days** | Numeric | Number of recorded hospitalization days during the three-year pre-index period. |
| **OMEQ - opioids** | Numeric | Total amount of dispensed OMEQ’s prescription opioids (ATC-code: N02A) during the three-year pre-index period. |
| **DDD - NSAIDs** | Numeric | Total amount of dispensed NSAIDs (ATC-code: M01A) DDD’s during the three-year pre-index period. |
| **DDD of other analgesics and antipyretics** | Numeric | Total amount of dispensed analgesics and antipyretics (mostly paracetamol) (ATC-code: N02B) DDD’s during the three-year pre-index period. |
| **DDD of other pain medication** | Numeric | Total amount of other pain medications than opioids, NSAIDs and paracetamol (ATC-code: tricyclic antidepressants [N06AA], SNRI [N06AX], gabapentin & pregabalin [N03AX12, N03AX16]) DDD’s during the three-year pre-index period. |
| **Type of LBP diagnosis** | Categorical | Type of LBP diagnosis at index (Dorsalgia, Intervertebral disc damage, Spinal Stenosis) |
| **Chronic pain related diagnosis** | Binary | If the patient had another chronic pain related diagnosis other than LBP diagnoses used for identification of the study population, during the three-year pre-index period. ICD-10 codes (taken from (Gustavsson et al., 2012) ):   - Cancer (C*) - Arthritis (M05-M08, M10-M15, M23-M25, M36, M77, R26) - Neuropathies (G50, G52-G64, G82, G97, M89, R29) - Multimorbidities (L89, L97-L98, M80) - Other back conditions (M43, M45-M46, M48-M49, M81-M82, M50-M51) - Headaches/migraine (G43-G44, R51) - Other conditions associated with chronic pain (F45, G96, M47, M53-M54, M70, M75, M79, M99, R07, R10, R52, S13, T85, T88, T92-T94) |
| **Death** | Binary | If the patients died during the post-index period. |

## Results

In Table S3 and S4, all BIC scores from each estimated model are presented. Table S3 presents for the pre-diagnosis groups and Table S4 for the post-diagnosis groups.

In the estimation, we assumed a censored normal distribution for the outcome variable (opioid dosing each month). Given that opioid use could not be negative, the low-end censoring number was set at 0 OMEQs and the high-end censoring number was set at an arbitrarily high number at 1,000,000 OMEQs (no patients had more than this). We did not consider time-varying effects, joint factors, or other patient characteristics, thus the GBTM was only based on opioid use during the specified time periods.

For a given number of pre-specified number of groups (between three and six), we ran all possible combinations of the trajectory shapes. Four trajectory shapes were considered for each group: only intercept, linear, quadratic, and cubic, which were denoted, respectively, 0, 1, 2, and 3 in the column ‘Model type’ in Table S3 and S4. The table entries are sorted by the BIC values (from best fit to worst fit, and starts in column a, then continues in column b and then c. Thus the models under column c have worse model fits than those in columns a and b. Normally, lower BIC values indicate better fit, but the BIC values reported in the Stata ‘traj’ package are scaled such that higher values closer to zero indicate better model fit.

From the first line of the left part in Table S3, we find that the model with six groups, where groups 1 and 2 were quadratic and groups 3-6 were cubic, represented by the sequence (2, 2, 3, 3, 3, 3), had the BIC score ‑815448. The star in Six* indicate that this model was the main model (highest BIC score) among all combinations with six groups. The best model fit for the model with five, four and three groups are also noted with a star.

Table S3: BIC values in estimated pre-diagnosis models.

|  | a |  |  | b |  |  | c |  |
| --- | --- | --- | --- | --- | --- | --- | --- | --- |
| # of groups | Model type | BIC values | # of groups | Model type | BIC values | # of groups | Model type | BIC values |
| Six* | 2, 2, 3, 3, 3, 3 | -815448 | Five | 1, 1, 2, 2, 3 | -850599 | Five | 0, 2, 2, 2, 3 | -879575 |
| Six | 1, 2, 3, 3, 3, 3 | -816984 | Five | 1, 1, 1, 2, 2 | -850602 | Five | 0, 0, 0, 2, 3 | -879981 |
| Six | 0, 2, 2, 3, 3, 3 | -819916 | Six | 0, 0, 0, 2, 2, 3 | -851800 | Four | 0, 1, 3, 3 | -880321 |
| Six | 0, 2, 2, 2, 3, 3 | -819993 | Five | 0, 1, 2, 3, 3 | -851818 | Four | 0, 1, 2, 3 | -880484 |
| Six | 0, 2, 2, 2, 2, 3 | -820626 | Five | 0, 1, 1, 2, 3 | -852037 | Four | 1, 1, 1, 1 | -880546 |
| Six | 0, 2, 2, 2, 2, 3 | -820626 | Five | 0, 1, 1, 3, 3 | -852042 | Five | 1, 1, 1, 1, 2 | -880569 |
| Six | 2, 2, 2, 3, 3, 3 | -822680 | Six | 1, 1, 1, 2, 2, 2 | -852485 | Five | 0, 0, 0, 1, 1 | -880761 |
| Six | 1, 1, 2, 2, 3, 3 | -824254 | Six | 1, 1, 2, 2, 2, 3 | -852591 | Four | 0, 1, 1, 2 | -881510 |
| Six | 1, 1, 2, 3, 3, 3 | -824451 | Five | 1, 1, 3, 3, 3 | -852730 | Four | 0, 1, 1, 3 | -881516 |
| Six | 1, 2, 2, 3, 3, 3 | -825794 | Five | 0, 1, 3, 3, 3 | -852864 | Five | 0, 1, 1, 2, 2 | -881534 |
| Six | 1, 1, 1, 2, 3, 3 | -826888 | Five | 1, 1, 1, 3, 3 | -853292 | Four | 0, 1, 1, 1 | -881712 |
| Six | 1, 1, 1, 1, 3, 3 | -826906 | Five | 1, 1, 1, 1, 3 | -853352 | Four | 0, 0, 3, 3 | -884606 |
| Six | 0, 0, 2, 3, 3, 3 | -828229 | Five | 1, 1, 2, 3, 3 | -853629 | Four | 0, 0, 2, 2 | -884824 |
| Six | 0, 1, 2, 2, 3, 3 | -828473 | Five | 1, 1, 1, 1, 1 | -853842 | Four | 0, 0, 2, 3 | -884830 |
| Six | 1, 3, 3, 3, 3, 3 | -828604 | Five | 0, 1, 1, 1, 3 | -855131 | Four | 0, 0, 1, 3 | -885593 |
| Six | 0, 1, 2, 2, 2, 2 | -828798 | Five | 0, 1, 1, 1, 2 | -855246 | Four | 0, 0, 1, 1 | -885746 |
| Six | 0, 1, 1, 1, 2, 2 | -828906 | Five | 0, 1, 1, 1, 1 | -855305 | Three* | 2, 3, 3 | -914581 |
| Five* | 2, 3, 3, 3, 3 | -830028 | Five | 0, 0, 2, 2, 3 | -858087 | Three | 2, 2, 2 | -914677 |
| Six | 1, 1, 2, 2, 2, 2 | -830031 | Six | 0, 0, 0, 3, 3, 3 | -859266 | Five | 2, 2, 2, 3, 3 | -914737 |
| Six | 2, 3, 3, 3, 3, 3 | -830058 | Four* | 2, 3, 3, 3 | -859282 | Three | 3, 3, 3 | -915006 |
| Six | 3, 3, 3, 3, 3, 3 | -830064 | Six | 0, 0, 0, 2, 3, 3 | -859596 | Five | 1, 2, 2, 2, 3 | -915786 |
| Six | 0, 0, 1, 2, 2, 2 | -830532 | Six | 0, 0, 0, 2, 2, 2 | -859611 | Three | 1, 3, 3 | -916400 |
| Six | 0, 0, 1, 2, 3, 3 | -830799 | Six | 0, 0, 0, 1, 2, 2 | -859641 | Three | 1, 2, 3 | -916578 |
| Six | 0, 1, 2, 2, 2, 3 | -831305 | Six | 0, 0, 0, 0, 2, 2 | -859712 | Three | 1, 1, 3 | -917999 |
| Five | 1, 3, 3, 3, 3 | -832310 | Six | 0, 0, 0, 1, 2, 3 | -859807 | Five | 0, 1, 2, 2, 2 | -927107 |
| Six | 1, 1, 1, 1, 2, 2 | -832383 | Six | 0, 0, 0, 0, 3, 3 | -859859 | Three | 1, 2, 2 | -930637 |
| Six | 1, 1, 1, 2, 2, 3 | -832446 | Six | 0, 0, 0, 1, 3, 3 | -860325 | Three | 0, 2, 3 | -931006 |
| Six | 1, 1, 1, 1, 2, 3 | -832672 | Six | 0, 0, 0, 1, 1, 3 | -860351 | Three | 0, 3, 3 | -931012 |
| Six | 0, 0, 1, 1, 2, 3 | -832681 | Four | 2, 2, 2, 3 | -860783 | Three | 0, 2, 2 | -931021 |
| Six | 1, 1, 1, 1, 1, 2 | -832841 | Four | 2, 2, 3, 3 | -860784 | Six | 0, 0, 0, 0, 2, 3 | -931280 |
| Six | 1, 2, 2, 2, 3, 3 | -833109 | Four | 1, 3, 3, 3 | -861661 | Three | 0, 1, 3 | -932834 |
| Six | 0, 0, 1, 2, 2, 3 | -833194 | Six | 0, 0, 0, 1, 1, 1 | -862399 | Three | 0, 1, 2 | -932850 |
| Six | 1, 1, 1, 1, 1, 3 | -838102 | Four | 2, 2, 2, 2 | -862669 | Four | 0, 1, 2, 2 | -932874 |
| Six | 1, 1, 1, 3, 3, 3 | -838443 | Five | 2, 2, 2, 2, 3 | -862715 | Three | 0, 1, 1 | -932973 |
| Six | 0, 1, 3, 3, 3, 3 | -838877 | Five | 0, 0, 2, 3, 3 | -862771 | Six | 0, 0, 0, 0, 1, 1 | -933464 |
| Five | 2, 2, 2, 2, 2 | -839326 | Four | 1, 2, 3, 3 | -863073 | Six | 0, 0, 0, 0, 1, 3 | -933636 |
| Six | 2, 2, 2, 2, 2, 2 | -839336 | Five | 0, 0, 2, 2, 2 | -863372 | Five | 0, 0, 0, 3, 3 | -933666 |
| Six | 2, 2, 2, 2, 2, 3 | -839356 | Five | 0, 0, 3, 3, 3 | -863376 | Five | 0, 0, 0, 3, 3 | -933666 |
| Six | 0, 1, 1, 3, 3, 3 | -839842 | Five | 0, 0, 1, 2, 3 | -863615 | Six | 0, 0, 0, 0, 0, 2 | -933684 |
| Five | 2, 2, 3, 3, 3 | -839867 | Five | 0, 0, 1, 3, 3 | -863620 | Six | 0, 0, 0, 0, 0, 3 | -933690 |
| Five | 1, 2, 3, 3, 3 | -840367 | Five | 0, 0, 1, 1, 3 | -864285 | Five | 0, 0, 0, 1, 3 | -933699 |
| Six | 0, 1, 1, 1, 2, 3 | -840478 | Four | 1, 1, 3, 3 | -864288 | Three | 1, 1, 1 | -933844 |
| Five | 1, 2, 2, 3, 3 | -840609 | Six | 1, 2, 2, 2, 2, 3 | -864568 | Four | 1, 1, 1, 2 | -933868 |
| Six | 0, 0, 1, 3, 3, 3 | -840733 | Four | 1, 2, 2, 2 | -864710 | Six | 0, 0, 0, 0, 1, 2 | -935442 |
| Five | 0, 3, 3, 3, 3 | -840757 | Four | 1, 2, 2, 3 | -864715 | Five | 0, 0, 1, 2, 2 | -936794 |
| Six | 0, 0, 3, 3, 3, 3 | -840813 | Five | 0, 1, 2, 2, 3 | -864727 | Five | 0, 0, 0, 0, 2 | -943436 |
| Six | 0, 1, 1, 1, 1, 3 | -840844 | Five | 1, 2, 2, 2, 2 | -864734 | Five | 0, 0, 0, 0, 3 | -943440 |
| Six | 0, 0, 1, 1, 3, 3 | -841970 | Four | 3, 3, 3, 3 | -865337 | Five | 0, 0, 0, 1, 2 | -943442 |
| Six | 0, 0, 1, 1, 2, 2 | -842119 | Six | 0, 0, 0, 1, 1, 2 | -865708 | Five | 0, 0, 0, 0, 0 | -943543 |
| Five | 0, 2, 2, 3, 3 | -842587 | Five | 0, 0, 1, 1, 2 | -866309 | Six | 0, 0, 0, 0, 0, 0 | -943555 |
| Six | 1, 2, 2, 2, 2, 2 | -843187 | Five | 0, 0, 1, 1, 1 | -866440 | Four | 0, 0, 0, 2 | -945236 |
| Six | 0, 1, 1, 1, 1, 1 | -844081 | Six | 0, 0, 1, 1, 1, 3 | -866469 | Four | 0, 0, 0, 3 | -945240 |
| Six | 0, 0, 1, 1, 1, 2 | -844535 | Four | 1, 1, 1, 3 | -867358 | Five | 0, 0, 0, 0, 1 | -945269 |
| Six | 0, 0, 1, 1, 1, 1 | -844672 | Five | 3, 3, 3, 3, 3 | -876742 | Four | 0, 0, 0, 1 | -945272 |
| Five | 1, 1, 2, 2, 2 | -845136 | Four | 0, 3, 3, 3 | -877493 | Six | 0, 0, 0, 0, 0, 1 | -945281 |
| Six | 0, 2, 2, 2, 2, 2 | -846086 | Four | 1, 1, 2, 3 | -877985 | Four | 0, 0, 0, 0 | -945353 |
| Five | 0, 2, 3, 3, 3 | -848348 | Six | 0, 1, 1, 1, 1, 2 | -878000 | Four | 0, 0, 1, 2 | -946497 |
| Six | 0, 1, 1, 2, 3, 3 | -848681 | Four | 0, 2, 3, 3 | -878383 | Three | 0, 0, 2 | -948910 |
| Six | 0, 1, 2, 3, 3, 3 | -848856 | Six | 2, 2, 2, 2, 3, 3 | -878440 | Three | 0, 0, 3 | -948916 |
| Five | 0, 2, 2, 2, 2 | -849333 | Four | 1, 1, 2, 2 | -879034 | Three | 0, 0, 1 | -949066 |
| Six | 1, 1, 3, 3, 3, 3 | -849455 | Four | 0, 2, 2, 2 | -879546 | Three | 0, 0, 0 | -949517 |
| Five | 1, 1, 1, 2, 3 | -849948 | Four | 0, 2, 2, 3 | -879551 | Three | 1, 1, 2 | -952726 |
|  |  |  |  |  |  | Three | 2, 2, 3 | -1019049 |
| *BIC values in estimated pre-diagnosis models. 0 indicate that one group was estimated with no intercept, 1 as linear, 2 as quadratic, and 3 as cubic. The star indicate the model with highest BIC value within the number of groups.* | | | | | | | | |

Table S4: BIC values in estimated post-diagnosis models.

|  | a |  |  | b |  |  | c |  |
| --- | --- | --- | --- | --- | --- | --- | --- | --- |
| # of groups | Model type | BIC values | # of groups | Model type | BIC values | # of groups | Model type | BIC values |
| Six* | 2, 2, 3, 3, 3, 3 | -1328971 | Five | 0, 3, 3, 3, 3 | -1376208 | Four | 1, 1, 2, 3 | -1427047 |
| Six | 2, 2, 2, 3, 3, 3 | -1331236 | Five | 1, 1, 2, 3, 3 | -1376753 | Four | 0, 2, 2, 2 | -1427425 |
| Six | 1, 2, 2, 3, 3, 3 | -1333892 | Five | 1, 1, 1, 1, 3 | -1377644 | Four | 0, 2, 2, 3 | -1427430 |
| Six | 1, 2, 3, 3, 3, 3 | -1333986 | Five | 1, 1, 1, 2, 3 | -1377694 | Five | 0, 2, 2, 2, 3 | -1427454 |
| Six | 1, 1, 2, 3, 3, 3 | -1335768 | Five | 1, 1, 1, 2, 2 | -1378152 | Six | 0, 2, 2, 2, 2, 3 | -1427478 |
| Six | 1, 1, 2, 2, 3, 3 | -1335770 | Five | 0, 1, 2, 3, 3 | -1378185 | Six | 0, 2, 2, 2, 2, 3 | -1427478 |
| Six | 2, 3, 3, 3, 3, 3 | -1339926 | Five | 1, 1, 2, 2, 2 | -1378716 | Four | 3, 3, 3, 3 | -1427771 |
| Six | 2, 3, 3, 3, 3, 3 | -1339926 | Six | 1, 1, 2, 2, 2, 3 | -1378746 | Four | 1, 3, 3, 3 | -1428015 |
| Six | 0, 2, 2, 3, 3, 3 | -1340425 | Six | 1, 2, 2, 2, 2, 3 | -1378752 | Four | 0, 0, 3, 3 | -1428225 |
| Six | 1, 1, 1, 3, 3, 3 | -1342403 | Five | 0, 1, 2, 2, 3 | -1378891 | Five | 0, 0, 1, 3, 3 | -1428612 |
| Six | 1, 1, 1, 1, 3, 3 | -1342598 | Six | 0, 0, 0, 1, 1, 3 | -1380201 | Five | 0, 0, 0, 3, 3 | -1429139 |
| Six | 1, 1, 1, 2, 2, 3 | -1343379 | Five | 0, 1, 1, 2, 3 | -1381232 | Five | 0, 0, 0, 3, 3 | -1429139 |
| Six | 1, 1, 1, 1, 2, 3 | -1343580 | Six | 0, 0, 0, 1, 2, 3 | -1382138 | Four | 0, 1, 3, 3 | -1429521 |
| Six | 1, 1, 1, 2, 3, 3 | -1344628 | Five | 0, 0, 1, 2, 2 | -1382958 | Four | 0, 1, 2, 2 | -1429789 |
| Six | 0, 1, 3, 3, 3, 3 | -1345043 | Five | 0, 1, 1, 2, 2 | -1383093 | Four | 0, 1, 2, 3 | -1429941 |
| Six | 1, 1, 1, 1, 2, 2 | -1345293 | Six | 0, 0, 1, 1, 3, 3 | -1383823 | Four | 2, 3, 3, 3 | -1430314 |
| Six | 0, 2, 2, 2, 3, 3 | -1358759 | Five | 0, 1, 3, 3, 3 | -1387828 | Four | 1, 2, 2, 3 | -1430957 |
| Six | 3, 3, 3, 3, 3, 3 | -1360327 | Five | 0, 1, 2, 2, 2 | -1388469 | Four | 0, 0, 2, 2 | -1431768 |
| Six | 3, 3, 3, 3, 3, 3 | -1360327 | Six | 0, 1, 2, 2, 2, 3 | -1388499 | Four | 0, 0, 2, 3 | -1431773 |
| Six | 0, 1, 2, 3, 3, 3 | -1360793 | Six | 0, 1, 1, 1, 1, 3 | -1391121 | Five | 0, 0, 2, 2, 3 | -1431797 |
| Six | 1, 2, 2, 2, 3, 3 | -1361769 | Five | 0, 0, 0, 1, 3 | -1392073 | Four | 0, 1, 1, 2 | -1432557 |
| Six | 0, 1, 2, 2, 3, 3 | -1362322 | Six | 0, 1, 1, 1, 2, 2 | -1392124 | Four | 0, 0, 0, 2 | -1432684 |
| Six | 0, 1, 2, 2, 2, 2 | -1363110 | Five | 0, 0, 1, 2, 3 | -1393153 | Six | 0, 0, 0, 0, 1, 2 | -1438650 |
| Six | 0, 0, 2, 3, 3, 3 | -1363153 | Six | 0, 0, 1, 2, 2, 3 | -1393181 | Six | 0, 0, 0, 0, 1, 3 | -1438655 |
| Six | 1, 1, 1, 1, 1, 3 | -1363834 | Six | 0, 0, 1, 1, 2, 2 | -1393247 | Six | 0, 0, 0, 0, 1, 1 | -1438719 |
| Six | 0, 1, 1, 2, 3, 3 | -1364348 | Five | 0, 0, 2, 3, 3 | -1397133 | Five | 0, 0, 0, 1, 2 | -1440471 |
| Six | 0, 1, 1, 3, 3, 3 | -1364354 | Six | 0, 0, 1, 1, 1, 2 | -1400688 | Five | 0, 0, 0, 1, 1 | -1440652 |
| Six | 0, 0, 3, 3, 3, 3 | -1364785 | Five | 1, 1, 1, 1, 2 | -1407758 | Six | 0, 0, 0, 1, 1, 2 | -1440676 |
| Six | 0, 0, 1, 2, 2, 2 | -1364902 | Five | 0, 0, 3, 3, 3 | -1409729 | Six | 0, 1, 1, 1, 1, 2 | -1446933 |
| Six | 0, 0, 1, 2, 3, 3 | -1365796 | Five | 1, 1, 1, 1, 1 | -1411036 | Five | 0, 0, 1, 1, 1 | -1449301 |
| Six | 0, 0, 1, 3, 3, 3 | -1365802 | Six | 0, 0, 1, 1, 1, 1 | -1413229 | Four | 1, 1, 1, 2 | -1451156 |
| Six | 0, 0, 0, 0, 3, 3 | -1366426 | Five | 0, 0, 2, 2, 2 | -1413831 | Four | 1, 1, 1, 3 | -1451161 |
| Six | 2, 2, 2, 2, 2, 2 | -1366475 | Six | 0, 0, 0, 1, 1, 1 | -1414010 | Six | 1, 1, 1, 1, 1, 2 | -1451484 |
| Six | 1, 3, 3, 3, 3, 3 | -1366627 | Six | 0, 0, 0, 2, 3, 3 | -1414256 | Four | 0, 0, 1, 2 | -1453383 |
| Six | 0, 0, 0, 3, 3, 3 | -1367020 | Six | 0, 0, 0, 1, 3, 3 | -1414459 | Four | 0, 0, 1, 3 | -1453389 |
| Five* | 3, 3, 3, 3, 3 | -1367483 | Five | 0, 0, 0, 0, 3 | -1414483 | Four | 0, 0, 1, 1 | -1453739 |
| Six | 0, 1, 1, 1, 2, 3 | -1367832 | Five | 0, 1, 1, 1, 3 | -1415798 | Five | 0, 0, 1, 1, 2 | -1453763 |
| Five | 2, 2, 3, 3, 3 | -1368063 | Five | 0, 1, 1, 1, 2 | -1416026 | Four | 0, 1, 1, 3 | -1454363 |
| Five | 2, 3, 3, 3, 3 | -1368069 | Six | 0, 0, 0, 2, 2, 2 | -1416234 | Four | 1, 1, 1, 1 | -1454686 |
| Six | 0, 0, 1, 1, 2, 3 | -1368139 | Six | 0, 1, 1, 1, 1, 1 | -1418032 | Four | 0, 1, 1, 1 | -1454729 |
| Six | 0, 0, 0, 0, 2, 3 | -1368332 | Five | 0, 0, 0, 2, 3 | -1418164 | Three* | 3, 3, 3 | -1499474 |
| Six | 1, 1, 2, 2, 2, 2 | -1368598 | Six | 0, 0, 0, 2, 2, 3 | -1418188 | Three | 2, 3, 3 | -1500831 |
| Five | 2, 2, 2, 3, 3 | -1368637 | Six | 0, 0, 0, 1, 2, 2 | -1418380 | Three | 1, 3, 3 | -1502069 |
| Six | 2, 2, 2, 2, 3, 3 | -1368661 | Five | 1, 1, 2, 2, 3 | -1419394 | Three | 2, 2, 3 | -1506671 |
| Six | 2, 2, 2, 2, 3, 3 | -1368661 | Six | 0, 0, 0, 0, 0, 3 | -1420882 | Three | 2, 2, 2 | -1506685 |
| Six | 0, 0, 0, 0, 2, 2 | -1368789 | Six | 0, 0, 1, 1, 1, 3 | -1421562 | Three | 1, 2, 3 | -1508061 |
| Six | 1, 1, 1, 2, 2, 2 | -1370671 | Four* | 2, 2, 3, 3 | -1421616 | Three | 0, 2, 3 | -1512630 |
| Five | 1, 3, 3, 3, 3 | -1371026 | Five | 0, 2, 2, 2, 2 | -1422199 | Three | 0, 0, 3 | -1513910 |
| Five | 1, 2, 3, 3, 3 | -1371268 | Four | 0, 3, 3, 3 | -1423287 | Three | 1, 1, 2 | -1522117 |
| Six | 0, 2, 2, 2, 2, 2 | -1371825 | Four | 2, 2, 2, 3 | -1423326 | Three | 1, 1, 3 | -1523830 |
| Five | 1, 2, 2, 3, 3 | -1371849 | Four | 2, 2, 2, 2 | -1424306 | Three | 1, 2, 2 | -1524731 |
| Six | 1, 2, 2, 2, 2, 2 | -1372711 | Five | 2, 2, 2, 2, 3 | -1424335 | Three | 1, 1, 1 | -1539147 |
| Six | 1, 1, 3, 3, 3, 3 | -1372918 | Five | 0, 0, 1, 1, 3 | -1424577 | Three | 0, 3, 3 | -1673516 |
| Five | 0, 2, 2, 3, 3 | -1373233 | Four | 1, 2, 2, 2 | -1424664 | Three | 0, 2, 2 | -1676093 |
| Five | 1, 1, 1, 3, 3 | -1374239 | Five | 1, 2, 2, 2, 3 | -1424694 | Three | 0, 1, 2 | -1699381 |
| Five | 1, 1, 3, 3, 3 | -1374435 | Five | 0, 1, 1, 1, 1 | -1424805 | Three | 0, 1, 3 | -1699387 |
| Five | 1, 2, 2, 2, 2 | -1374809 | Four | 1, 1, 3, 3 | -1424993 | Three | 0, 1, 1 | -1699589 |
| Five | 2, 2, 2, 2, 2 | -1375243 | Four | 1, 2, 3, 3 | -1425483 | Four | 0, 0, 0, 3 | -1707963 |
| Five | 0, 1, 1, 3, 3 | -1375689 | Six | 2, 2, 2, 2, 2, 3 | -1426148 | Six | 0, 0, 0, 0, 0, 0 | -1707972 |
| Five | 0, 2, 3, 3, 3 | -1375959 | Six | 2, 2, 2, 2, 2, 3 | -1426148 | Four | 0, 0, 0, 1 | -1708061 |
|  |  |  | Four | 0, 2, 3, 3 | -1426799 | Five | 0, 0, 0, 0, 1 | -1708073 |
|  |  |  | Four | 1, 1, 2, 2 | -1427044 | Six | 0, 0, 0, 0, 0, 1 | -1708085 |
|  |  |  |  |  |  | Four | 0, 0, 0, 0 | -1708111 |
|  |  |  |  |  |  | Five | 0, 0, 0, 0, 0 | -1708123 |
|  |  |  |  |  |  | Five | 0, 0, 0, 0, 2 | -1708135 |
|  |  |  |  |  |  | Six | 0, 0, 0, 0, 0, 2 | -1708146 |
|  |  |  |  |  |  | Three | 0, 0, 2 | -1713165 |
|  |  |  |  |  |  | Three | 0, 0, 1 | -1713405 |
|  |  |  |  |  |  | Three | 0, 0, 0 | -1714458 |
| *BIC values in estimated post-diagnosis models. 0 indicate that one group was estimated with no intercept, 1 as linear, 2 as quadratic, and 3 as cubic. The star indicate the model with highest BIC value within the number of groups.* | | | | | | | | |

Figure S2 presents the patterns of opioid use across the models estimated using GBTM. The panel names refer to the number of pre-specified groups the model. The exact models presented are the models with the best BIC values (indicated with stars in Table S3 and S4) for each number of pre-specified groups. In each panel, the curves to the left indicate the pre-diagnosis groups and the curves to the right the post-diagnosis groups. Thus, in the panel with three groups, the curves to the left are the opioid use trajectories for the model with three groups with the model combinations (2, 2, 3) and BIC value of -914581.

| 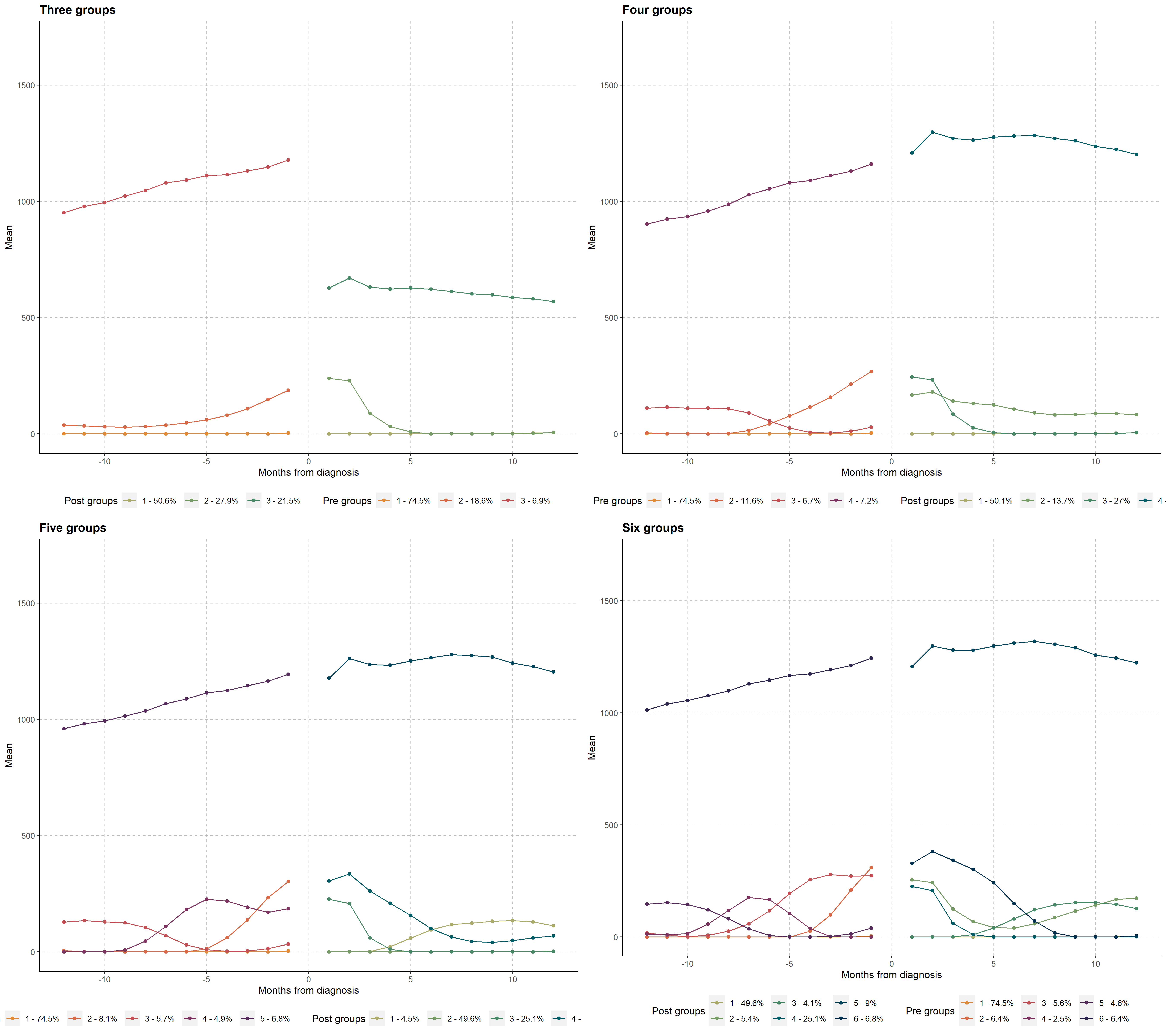  Figure S2: Trajectories of all GBTM estimated groups, stratified by the number of pre-specified number of groups. The chosen trajectories are best on the models with the best model fit according to BIC values. |
| --- |

Figure S3 presents Kaplan-Meier plots from the time of diagnosis to death stratified by the pre-diagnosis groups (with three groups). Notice the low number of events and the Y axis

| 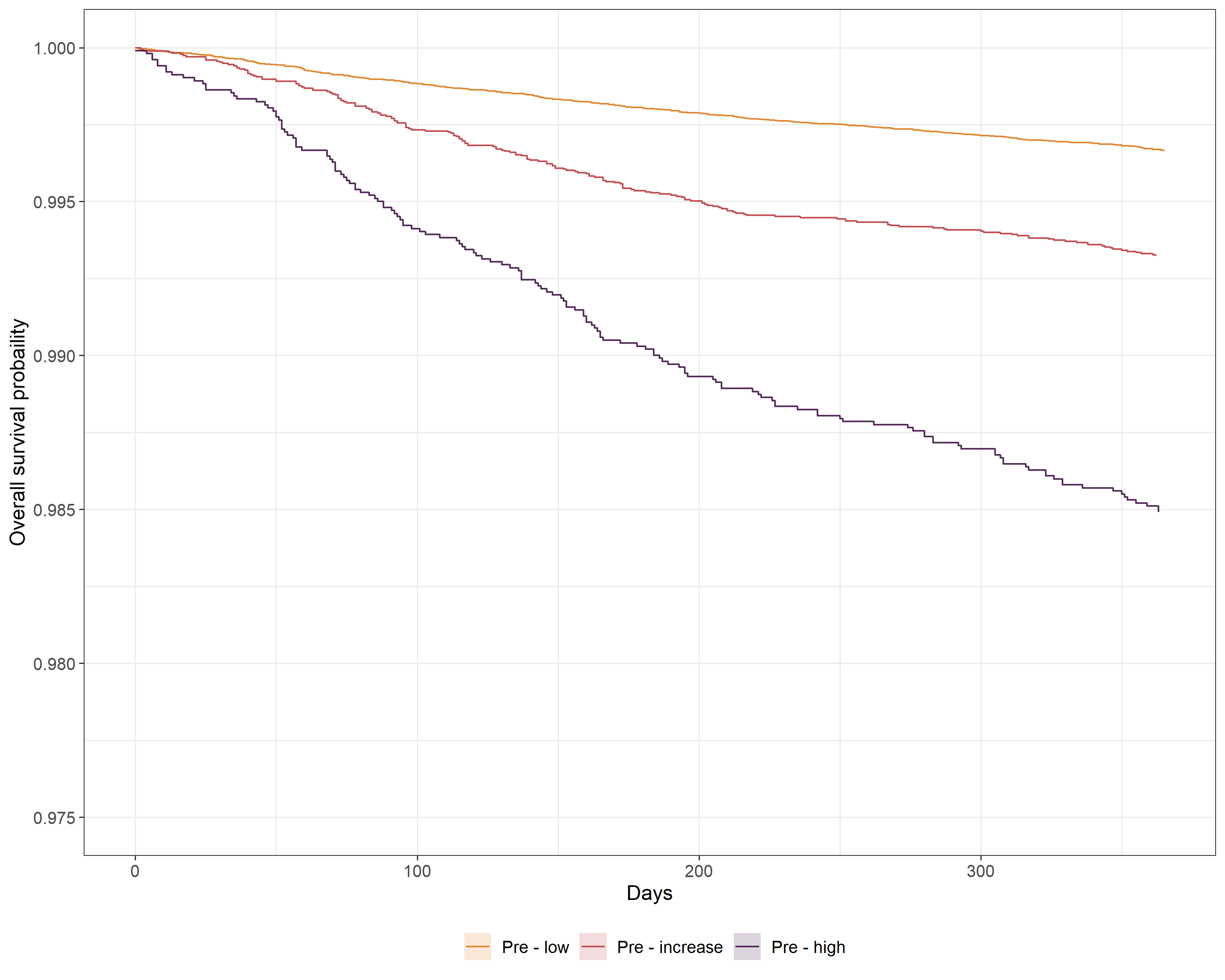  Figure S3: Survival from diagnosis by pre-diagnosis trajectory groups. |
| --- |

**References**

Gustavsson A, Bjorkman J, Ljungcrantz C, Rhodin A, Rivano-Fischer M, Sjolund KF, Mannheimer C. Socio-economic burden of patients with a diagnosis related to chronic pain--register data of 840,000 Swedish patients. Eur J Pain 2012;16: 289-299.

Svendsen K, Skurtveit S, Romundstad P, Borchgrevink PC, Fredheim OMS. Differential patterns of opioid use: Defining persistent opioid use in a prescription database. European Journal of Pain 2012;16: 359-369.

The Association for the publication of the Norwegian Drug Handbook. Tabell 1: Ekvianalgetiske doser av opioidanalgetika og virkningstid [equianalgetic doses of opioid analgesics and time of effect] 2019; Available from: <https://www.legemiddelhandboka.no/L20.2/Tabeller>.

The Danish Health Data Authority. Forbruget af opioider er aftaget efter øget fokus på brugen. 2020; Available from: <https://sundhedsdatastyrelsen.dk/-/media/sds/filer/find-tal-og-analyser/laegemidler/smertestillende-medicin/forbrug_af_opioider_2019.pdf>.
